# Shape Morphable Hydrogel/Elastomer Bilayer for Implanted Retinal Electronics

**DOI:** 10.3390/mi11040392

**Published:** 2020-04-09

**Authors:** Muru Zhou, Do Hyun Kang, Jinsang Kim, James D. Weiland

**Affiliations:** 1 Macromolecular Science and Engineering, University of Michigan, Ann Arbor, MI 48109, USA; muru@umich.edu; 2 Department of Materials Science and Engineering, University of Michigan, Ann Arbor, MI 48109, USA; kangdh@umich.edu; 3 Department of Chemical Engineering, University of Michigan, Ann Arbor, MI 48109, USA; 4 Department of Biomedical Engineering, University of Michigan, Ann Arbor, MI 48109, USA; 5 Department of Chemistry, University of Michigan, Ann Arbor, MI 48109, USA; 6 Biointerfaces Institute, University of Michigan, Ann Arbor, MI 48109, USA; 7 Department of Ophthalmology and Visual Sciences, University of Michigan, Ann Arbor, MI 48109, USA

**Keywords:** hydrogel, bilayer, responsive materials, retinal prosthesis, shape memory materials

## Abstract

Direct fabrication of a three-dimensional (3D) structure using soft materials has been challenging. The hybrid bilayer is a promising approach to address this challenge because of its programable shape-transformation ability when responding to various stimuli. The goals of this study are to experimentally and theoretically establish a rational design principle of a hydrogel/elastomer bilayer system and further optimize the programed 3D structures that can serve as substrates for multi-electrode arrays. The hydrogel/elastomer bilayer consists of a hygroscopic polyacrylamide (PAAm) layer cofacially laminated with a water-insensitive polydimethylsiloxane (PDMS) layer. The asymmetric volume change in the PAAm hydrogel can bend the bilayer into a curvature. We manipulate the initial monomer concentrations of the pre-gel solutions of PAAm to experimentally and theoretically investigate the effect of intrinsic mechanical properties of the hydrogel on the resulting curvature. By using the obtained results as a design guideline, we demonstrated stimuli-responsive transformation of a PAAm/PDMS flower-shaped bilayer from a flat bilayer film to a curved 3D structure that can serve as a substrate for a wide-field retinal electrode array.

## 1. Introduction

Flexible and soft shape-morphing materials undergo shape transformation when responding to certain stimuli and have gained an increasing interest in both research and industry [1,2,3]. The hybrid bilayer is one feasible and promising approach to create a soft, shape-changing material. A hybrid bilayer consists of a responsive layer, which can contract or expand when responding to a given stimulus, and a passive layer, which constrains the motion of the responsive layer. Torsion, bending, and buckling can be generated by the residual stress and eventually lead to the shape transformation from a two-dimensional (2D) to a three-dimensional (3D) structure [4,5]. Hybrid bilayers have various applications including cell encapsulation [6,7,8], drug delivery [9], micro robots [10], actuators [11,12,13], and flexible electronics [14,15,16,17,18].

Metallic bilayer actuators [19,20] have a long history in sensing, but exhibit small shape changes and usually are responsive only to temperature change. In contrast, hydrogels can respond to various stimuli, including temperature, pH, light, chemicals, and electrical field [21] and have a larger extent of shape change [8]. When hydrogel is constrained by the passive layer with a different swelling property, the swell or de-swell of the hydrogel can induce internal residual stress and result in shape transformation. The transformed shape is determined by the mechanical properties of each layer, the thickness ratio between the two layers, and also the dimensions and size of the bilayer. Studying how those parameters affect the final shape can help to design the shape transformation precisely and rationally. Kim et al. have studied the shape transformation of a hydrogel bilayer with different thickness ratios between the two layers [22]. Stoychev et al. have reported that the folding behavior varies with the aspect ratio of the hydrogel bilayer [23]. However, less research has focused on varying the intrinsic mechanical property of hydrogel to tune the transformed shape. Intrinsic properties of hydrogel can be modulated by varying the initial monomer concentration [24], cross-link density [25], initiator concentration [26] of the pre-solution, and also the ultraviolet (UV) intensity for photo-curing [27]. Decoding the relationship between intrinsic properties of hydrogel and the 3D structure of the bilayer can help to guide bilayer fabrication to achieve the ideal shape transformation, as a necessary first step towards more complicated designs and applications. For example, the bilayer has the potential to be used as a substrate for epiretinal prosthesis. Current retinal electrode arrays stimulate only the central retina, due to the size limit of the array for implantation [28]. A compact array that can gradually unfold to a desired curvature to reach peripheral retinal areas will expand the visual field of the retinal implant patient [29,30,31,32,33]. The potential application of the bilayer for epiretinal electrode arrays requires the fine-tuning of the programed 3D structure.

Here, we investigated the effect of intrinsic properties of hydrogel on the transformed shape using a hydrogel/elastomer bilayer system responsive to moisture. The findings from this study will provide design guidance for a wide-field epiretinal electrode array. The responsive part of the bilayer consists of polyacrylamide (PAAm), a biocompatible, and soft hydrogel. Polydimethylsiloxane (PDMS) acts as the passive layer because PDMS is a common soft electrode array substrate. Benzophenone is chosen as a bonding promoter to prevent delamination between the two layers [34]. First, we fabricated the hydrogel/elastomer bilayer with different PAAm hydrogel properties by varying the initial monomer concentration in the pre-solution of PAAm hydrogel while keeping the same cross-linker ratio, the initiator ratio, and the UV light intensity. The robust bilayers transformed from a flat to a curved structure in water without delamination. We found that the curvature of the bilayer increased with the initial monomer concentration of the pre-solution. A simple model agrees with the experimental data well and provides theoretical validation of our design. This result can help us to formulate the hydrogel pre-solution to achieve the desired curvature. Guided by the relationship between the intrinsic properties of hydrogel and the final shape, we demonstrated a flower-shaped bilayer for future retinal electrode array applications.

## 2. Materials and Methods 

### 2.1. Materials

Poly(N-isopropylacrylamide) (PNIPAAm; Sigma–Aldrich A3553; Mn ~40,000, Sigma–Aldrich, St. Louis, MO, USA), acrylamide (AAm; Sigma–Aldrich A3553; ≥99%), 2-hydroxy-2-methylpropiophenone (Irgacure 1173; Sigma–Aldrich 405655; 97%), N, N′-Methylenebisacrylamide (MBAM; Sigma–Aldrich M7279; ≥99.5%) and benzophenone (Sigma–Aldrich B9300; ≥ 99%) were purchased from Sigma Aldrich. Polydimethylsiloxane (PDMS; Dow Corning Sylgard 184 Silicone Elastomer Kit, Dow Corning, Midland, MI, USA) was purchased from Dow Corning. PAAm hydrogel layer was molded from a Teflon mold (65 mm × 40 mm × 0.1 mm (length × width × thickness)).

### 2.2. Fabrication of PDMS Elastomer

PNIPAAm was used as the sacrificial layer between the PDMS and the glass slide. 10 mg/mL PNIPAAm in ethanol was spin-coated on glass slides with a spin speed of 4000 rpm for 60 s, using a spin coater (Solitec Spinner, Solitec Wafer Processing Inc., Milpitas, CA, USA) at the Lurie Nanofabrication Facility (LNF) at the University of Michigan. Then, the glass slides were heated at 80 °C for 90 s to evaporate the excess solvent [35]. PDMS prepolymer and its curing agent (10:1 weight ratio) was mixed for 2 min and degassed in a vacuum desiccator for 40 min. The mixture was carefully poured on the glass slide to avoid bubbles and spin-coated with a spin speed of 250 rpm for 30 s. The PDMS was cured at 80 °C for 2 h on a hotplate. After curing, the PDMS was patterned with a laser (HL40-5g “Hobby Advanced”, Full Spectrum Laser, Las Vegas, NV, USA) into rectangular strips (17 mm × 2.5 mm (length × width)) for later release.

### 2.3. Fabrication of PDMS/Hydrogel Bilayer

Cured PDMS was immersed into benzophenone solution (10 wt% in ethanol) for 4 min. Then, PDMS was washed with methanol three times and dried in air. Different amounts of acrylamide (AAm) were dissolved in water to form pre-solutions with acrylamide wt% in the range of 5% to 20%. The cross-linker ratio (the weight ratio of MBAM to AAm) was fixed at 3.89%, and the initiator ratio (the weight ratio of Irgacure 1173 to AAm) was fixed at 0.356%. The pre-gel solution was poured into the Teflon mold and covered by the PDMS elastomer. The pressure from the PDMS insured the even distribution of the pre-solution in the mold. The pre-gel solution was cured under an ultraviolet lamp (ML-3500C, Spectroline, Westbury, NY, USA) for 5 min. After curing, the slides were immersed in deionized (DI) water at room temperature, and the bilayer strips were released. For the fabrication of the flower-shaped bilayer, the hydrogel covered only the petals, but not the center area, with a diameter of 3.8 mm.

### 2.4. Mechanical Test

Tensile tests of PDMS and PAAm hydrogel were completed with Texture Analyzer (TA. XT plusC, Stable Micro Systems, Surrey, UK) at Van Vlack Lab at the University of Michigan. The testing strain speed used for PAAm hydrogel was 0.1 mm/s, and for PDMS was 0.5 mm/s. The testing samples were fabricated into a dog bone shape using an acrylic mold (gauge width 11 mm, gauge length 40 mm). The PAAm hydrogel dog bone had a thickness of 7 mm and the PDMS dog bone had a thickness of 0.3 mm. The Young’s modulus data were calculated from the stress–strain curve of the hydrated hydrogel. The expansion strain of PAAm hydrogel was defined as the ratio of the difference in lengths between dried and swollen states to the length of the dried state ($expansion\;strain=\frac{length_{\mathrm{hydrated}\;}-\;length_{\mathrm{dried}}}{length_{\mathrm{dried}}})$. The samples were immersed in ethanol overnight to de-swell, then put in an oven at 40 °C for 10 min to evaporate the ethanol thoroughly. After measuring the dimensions, the dried PAAm hydrogel was immersed in DI water overnight to swell and the dimensions were measured again to calculate the expansion strain.

### 2.5. Image Analysis

Bilayer strips were put into DI water at room temperature until reaching stable configurations. A camera (D7200, Nikon, Tokyo, Japan) was used to take photos of the hydrated hydrogels, and the curvature analysis was done using the digital imaging processing software Fiji and the plugin Kappa. Kappa measured the curvature of each point on the bilayer spline, then the average of the curvature was used to represent the curvature of each bilayer strip. For imaging the morphology and adhesion of the bilayer interface, bilayer strips were pre-frozen in liquid nitrogen for 5 min and then freeze-dried for 24 h. The samples were imaged by scanning electron microscopy (SEM; TESCAN RISE, TESCAN, Brno, Czech Republic) at Michigan Center for Materials Characterization (MC2) at the University of Michigan. To get the thickness input for the computational simulation, the bilayer strips were dried using ethanol with the method mentioned above. Then, the fully dehydrated bilayer strips were imaged with SEM to determine the thickness of PAAm hydrogel and PDMS. The input PDMS thickness was set as 300 μm, and the PAAm hydrogel thickness was normalized according to the actual PDMS thickness as: 74.5 μm (5 wt%), 77.9 μm (7 wt%), 87.7 μm (10 wt%), 96.4 μm (15 wt%), and 196.1 μm (20 wt%).

## 3. Results

### 3.1. Fabrication of Hydrogel Bilayer

**Figure 1:**
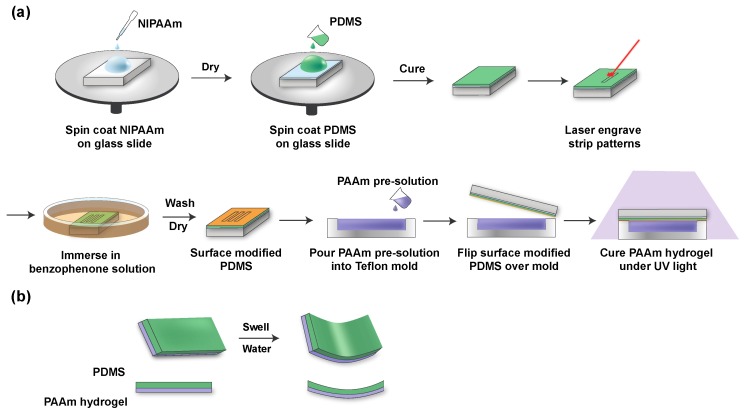
(**a**) Schematic illustration of the fabrication of the hydrogel/elastomer bilayer. (**b**) Mechanism of the shape transformation of the bilayer strip. Images not to scale.

PDMS layers less than 120 μm were fragile and difficult to handle without tearing. To ensure both the flexibility and durability for future use as a retinal electrode array, PDMS thickness was set to 300 +/− 50 μm [36]. The fabrication scheme is shown in Figure 1a. First, the PNIPAAm solution was spin-coated on glass slides as a sacrificial layer, based on PNIPAAm’s dissolution in water lower than 32 °C. Then, the PDMS prepolymer mixture was spin-coated on top of the PNIPAAm-coated glass slides and cured. The thickness of PDMS decreased as the spin speed and spin time increased. We noted that a spin speed at 250 rpm for 30 s could produce a PDMS layer with a thickness around 300 μm. After curing, the PDMS was cut by laser into rectangular strips (17 mm × 2.5 mm (length × width)). Next, surface modification was done by immersing the slide into the benzophenone solution for 4 min, followed by washing three times with methanol and drying with air. Hydrogel pre-solution of varying monomer concentrations was poured into a Teflon mold, and surface-modified PDMS attached to the glass slide was flipped onto the mold. After curing with the UV irradiation for 5 min, the glass slides were immersed into DI water to release the bilayer strips quickly. Released bilayer strips curved in water instantly (Figure 1b).

### 3.2. Initial Monomer Concentration Is Proportional to Final Bilayer Curvature

**Figure 2:**
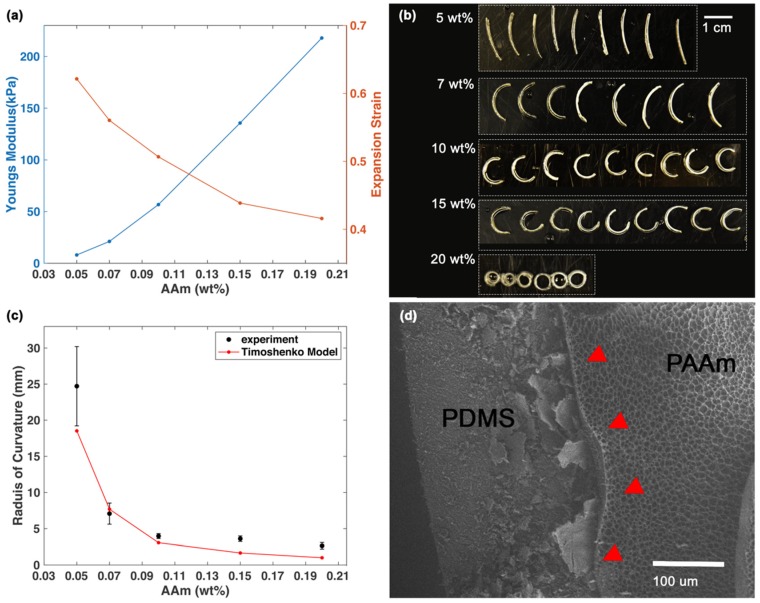
Characterizations of hydrogels and curvature measurement of bilayer strips. (**a**) Young’s modulus and expansion strain of hydrated hydrogels with different AAm initial concentrations. (**b**) Hydrated bilayer strips (17 mm × 2.5 mm (length × width)) with different AAm wt%. Images were captured with samples on a dry surface to allow consistent arrangement of the samples, but the dry environment slightly affected the curvature. The images used for curvature analysis were taken with the samples in deionized (DI) water. The scale bar is 1 cm. (**c**) The radius of curvature of bilayer strips with different initial monomer concentrations. The black dots represent experimental data. The sample size is six for bilayer with 20 wt% AAm and nine for other concentrations. The error bar represents standard deviation. The red line represents simulation results from Timoshenko model. (**d**) SEM image of the bilayer interface (red arrows) with 10% wt% AAm initial monomer concentration. This sample underwent swelling, then freeze-drying. The scale bar is 100 μm. The contrast was enhanced (Adobe Photoshop).

To explore how the intrinsic properties of hydrogel affect the bilayer curvature, we first investigated the intrinsic properties of PAAm hydrogel with different initial monomer concentrations. Prior work has shown that a higher initial monomer concentration led to a decreased swelling ratio and a higher modulus of hydrogel [24,37,38]. A shown in Figure 2a, we found that with a high initial monomer concentration, the PAAm hydrogel had (1) a decreased expansion strain, (2) an increased Young’s modulus when hydrated, and (3) an increased thickness when fully dried. Preparing hydrogel in a dilute pre-gel solution with low monomer concentration leads to ineffective usage of cross-linkers and a reduced cross-link density. At the same time, the resulting polymer concentration of the formed gel also decreased, which led to a decreased thickness in the dried hydrogel. While the elastic modulus is related to the effective cross-link density, the modulus was expected to reduce with a low initial monomer concentration. Meanwhile, the reduced polymer concentration allows the PAAm gel to disentangle more easily during hydration, effectively increasing the swelling ability [24,39]. Notably, the highest modulus of PAAm hydrogel (20 wt% initial monomer concentration) was 217.86 kPa, which remained lower than that of PDMS (562.40 kPa). Therefore, hydrogel was softer and presumably more tissue compatible than PDMS.

When constrained by a non-swelling passive layer, the responsive layer of the hydrogel can only swell asymmetrically. The tensile stress on PDMS and the compressive stress on PAAm hydrogel induced bending moments, producing a curved structure with PDMS on the concave side. We investigated the curvature of bilayer strips with five different initial monomer AAm concentrations: 5, 7, 10, 15, 20 wt%. At the same time, we kept constant the molding conditions, cross-linker ratio (the weight ratio of MBAM to AAm), and the initiator ratio (the weight ratio of Irgacure 1173 to AAm). We noted that with high initial monomer concentration, the curvature of the bilayer increased (Figure 2b,c). Because the radius of curvature is the reciprocal of the curvature, the radius of curvature decreased with a higher monomer concentration. 

The bimetallic strip model developed by Timoshenko [40] and its derivatives are widely used to explain the mechanical behaviors of the hydrogel bilayer strip [22,41,42]:

(1) $$\frac{1}{\rho }=\frac{6\times \varepsilon_{PAAm}\times {\left(1+m\right)}^{2}}{h(3{\left(1+m\right)}^{2}+\left(1+mn\right)\left(m^{2}+\frac{1}{mn}\right))}$$

where ρ is the radius of curvature at a given time, $\varepsilon_{PAAm}$ is the expansion strain of PAAm hydrogel, $\varepsilon_{PAAm}=\frac{∆l}{l}$. Here, ∆l is the difference of dimensions of hydrogel between dried state and hydrated state, and l is the dimensions of the hydrogel in the dried state. $m=\frac{h_{PDMS}}{h_{PAAm}}$ is the thickness ratio between the two layers. h is the total thickness of the bilayer. $n=\frac{E_{PDMS}}{E_{PAAm}}$ is the ratio of Young’s modulus of two layers. 

The radius of curvature is related to the expansion strain, the Young’s modulus, and the thickness ratio of the bilayer, as shown in Equation (1) [40]. Changing the monomer concentration will change all of these parameters in ways that will have opposing effects on the radius of the curvature. The decreased expansion strain of the hydrogel layer leads to a decrease in the curvature, while the increased Young’s modulus and thickness of the hydrogel leads to a larger curvature. Shown in Figure 2c, we used Equation (1) as the model and input the mechanical data and thicknesses of the two layers. The predicted and experimental curvature follow a similar trend.

The SEM image (Figure 2d) of the freeze-dried sample shows a robust interface between PAAm hydrogel and PDMS. The monomers together with cross-linkers formed a network of porous PAAm hydrogel, similar to previously reported work [10,43] The relationship between the initial monomer concentration and the transformed curvature can help us to design the bilayer formula to achieve a desired curvature.

### 3.3. PAAm/PDMS Bilayer Can Be Retinal Array Substrate

**Figure 3:**
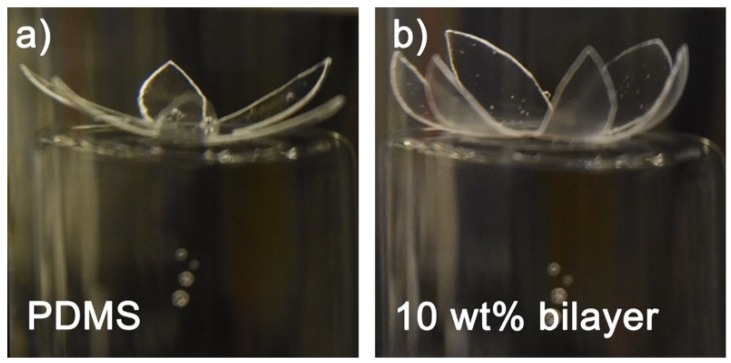
(**a**) Polydimethylsiloxane (PDMS) without polyacrylamide (PAAm) layer. (**b**) Bilayer samples with 10 wt% initial monomer concentration. All samples in DI water. The flower shape is designed to unfold after implantation in the eye. This structure shown would cover 34 mm on the retina, equivalent to a visual field of about 113° [46].

We further investigated the potential of the bilayer to be used as the substrate for a wide-field epiretinal prosthesis. Given the importance of peripheral vision with respect to mobility [44,45], our design will provide visual perception over 100° of the visual field. We fabricated a six-petal flower-shaped bilayer with a diameter of 34 mm by means of the same technique shown in Figure 1a. If we assume that a human eye is a perfect sphere with a radius of curvature of 11.5 mm and 1° of visual field is equal to 300 μm on the retina [46,47], the Argus II electrode array (6.19 mm diagonally) requires a 31° bending and can restore approximately 21° of visual field, while our bilayer with a diameter of 34 mm requires a 169° bending and can restore 113° of visual field. From Figure 3, we demonstrated that PDMS without hydrogel had negligible curvature, while the bilayer flower had a curved shape because of the swelling of the PAAm hydrogel. Based on PDMS and PAAm hydrogel thickness, the bilayer with a 34 mm diameter can be rolled into a spiral tube with a theoretical diameter of 1.58 mm. The bilayer can be fixed by drying and would expand once implanted. Argus II requires a surgery with a 5-mm incision in the eye wall to insert the retinal electrode array [28]. Our rolled bilayer will easily pass through a 5 mm incision and may allow the incision size to be reduced. 

## 4. Discussion

In this study, we demonstrated the design of a robust hydrogel/elastomer bilayer system that can transform from 2D to 3D after immersion in water. Spin-coating and molding fabrication methods are used to achieve reproducible thickness control and bending behaviors. We investigated the effect of the initial monomer concentration of hydrogel pre-solution on the curvature of the transformed shape. The expansion strain, Young’s modulus, and the thickness ratio between the two layers all contributed to the final curvature. A higher monomer concentration in the pre-solution produced a larger curvature and a smaller radius of curvature. While the expansion strain of hydrogel decreased as the monomer concentration increased, the enlarged modulus and thickness of the hydrogel had a dominant effect and consequently produced a larger bending motion. Swelling was performed in DI water. The aqueous environment in the eye after vitrectomy is similar to saline (NaCl) [28]. NaCl does affect the swelling properties of PAAm hydrogel, but to a relatively small degree at the physiological concentration [48]. As we get closer to in-vivo testing, we will recalibrate the bilayer curvature (Figure 1) in physiological saline. Timoshenko’s model provides a theoretical basis for the effect of varying initial monomer concentration on the bilayer curvature. The effect of the aspect ratio and thickness ratio on the transformed curvature have also been reported [22,23]. Although those factors could all effectively tune the curvature, our application of a retinal prosthesis required a relatively thin layer of PDMS and a fixed-shape design. Therefore, varying initial monomer concentration is more feasible and practical to precisely adjust the transformed curvature.

**Figure 4:**
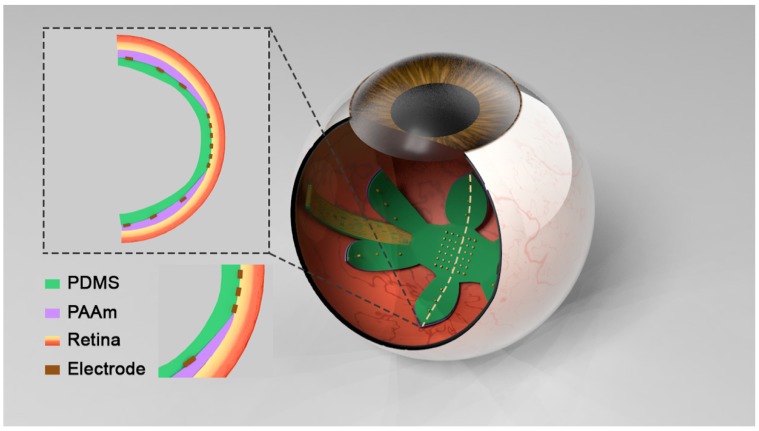
Conceptual illustration of the wide-field bilayer array implanted in the eye. Inserted picture demonstrates the cross-section of the interface between the array and the retina.

With the understanding of how the intrinsic mechanical properties of hydrogel affect the final shape, we were able to rationally design the hydrogel formula to make a 3D shape with the desired curvature (Figure 3). Figure 4 illustrates a concept for incorporating these design features into a wide-field epiretinal prosthesis. In our proposed design, the epiretinal prosthesis uses PDMS for the multielectrode array substrate and insulation layers. Conducting lines will be gold (Au), with additional adhesion layers as needed—for example, chromium (Cr). Our electrode array design is expected to have 100 electrodes in the central area with a diameter of 200 μm and up to four electrodes on each peripheral petal [49]. To enable chronic stimulation, electrodes can be electrodeposited platinum [50] or platinum–iridium [51]. To facilitate pattern stimulation, electrodes are denser and smaller in the central area. Peripheral electrodes are more sparse and larger, since their role is to alert the user to objects in the periphery. Even though the petals of the proposed array will not cover the entire peripheral retina, the accompanying camera and video processing system can be programmed to map continuous regions of the peripheral camera field-of-view to each of the petals. Central electrodes will have a lower allowable stimulus charge due to their small size [52]. Thus, the central electrodes must be close to the retina for low perceptual thresholds [53], and hydrogel will not be on the central part of the array, since the hydrogel layer separates the electrodes from the retina. A hydrogel layer outside the central region will ensure the curvature of the array, which will also help the central electrodes be in close retinal contact. The hydrated hydrogel in DI water has a comparable conductivity to DI water [54]; therefore, the large peripheral electrodes can still pass stimulus currents through the hydrogel into the retina. Although separating the peripheral electrodes from the retina will increase the perceptual threshold, large peripheral electrodes will safely pass more currents to overcome this issue. This bilayer system can be used in other neural or biological interfaces requiring a 3D anatomical interface and soft materials as well. 

Other approaches to wide-field array have been demonstrated, including foldable arrays made of polyimide [30], parylene C [33], PDMS [32], and shape-memory polymers (SMP) [31]. Most wide-field arrays utilized the elasticity of the substrate or manipulation by the surgeon for the unfolding of the array. The SMP bilayer wide-field array is similar to our work and the two approaches share the advantage of unfolding to a pre-determined curvature. However, the unfurling time of the SMP bilayer was less than one minute. This unfolding can be sudden and unpredictable, which may lead to unintended contact with the retina. In contrast, a hydrogel bilayer approach allows slow, controlled unfolding as the functional polymer layer changes shape. Furthermore, the SMP bilayer array used polyimide as the electrode array substrate, which is significantly stiffer than PDMS and hydrogel. The hydrogel-coated array has a significantly lower modulus to better conform to the curvature of the retina. Since curvature mismatch will result in pressure on the retina, the hydrogel/elastomer wide-field array will be less damaging to the retina in this scenario. 

**Figure A1:**
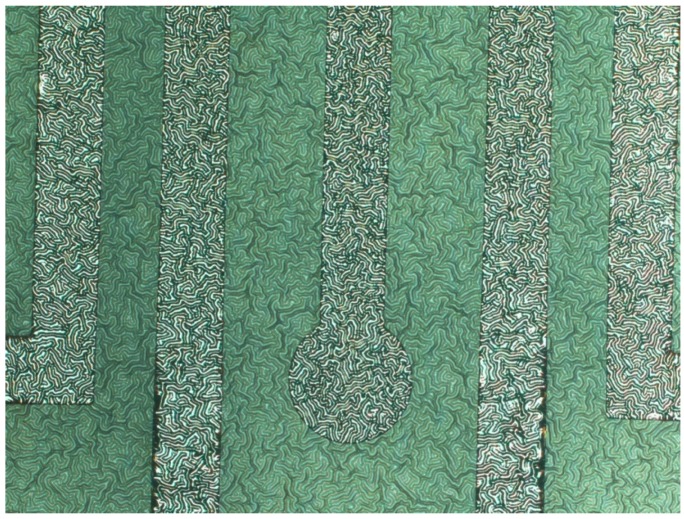
Cr/Au/Cr metal layer deposited on PDMS. Conductive tracks were designed to be 100 μm in width and the circular electrode to be 200 μm in diameter.

In the follow-up work, we will use the PAAm/PDMS bilayer as the substrate and will deposit metal electrodes onto it. Prior works have shown the possibility of fabricating electrodes on PDMS [36,55,56]. Initial fabrication based on the procedure in [55] provided successful deposition of a Cr/Au/Cr metal layer on PDMS (Figure A1). Importantly, stretching the metal lines was plausible owing to the micro-wrinkles in the metal film. Flexible and stretchable metal leads are necessary when making an electrode array that has a small form factor for minimally invasive implantation followed by unfolding in the vitreous body after implantation. A prior study further demonstrated that a thin layer of metal on PDMS had an improved stretchability [57], due to micro-cracks in the layer. It was proposed that the micro-cracked layer formed a network that maintained electrical continuity under stretching. We will further systematically investigate the metal electrode deposition and the feasible extent of folding in due course.

## 5. Conclusions

We demonstrated a hydrogel-PDMS bilayer structure that curves in a predictable manner when immersed in water. Initial monomer concentration was directly related to the resulting curvature. Our planned application of this technology is a wide-field retinal prosthesis, but this bilayer technology has applicability to other neural interface or biosensor systems that require a compliant 3D interface.
